# Supplementary material for: Influence of the Soluble–Insoluble Ratios of Cyclodextrins Polymers on the Viscoelastic Properties of Injectable Chitosan–Based Hydrogels for Biomedical Application
Source: Polymers (Basel). 2019 Jan 26;11(2):214. doi: 10.3390/polym11020214 (PMC6419078; doi:10.3390/polym11020214)
Supplement: Supplementary file 1 [file polymers-11-00214-s001.pdf]

Supplementary Materials

Influence of the Soluble–Insoluble Ratios of Cyclodextrins Polymers on the Viscoelastic Properties of Injectable Chitosan–Based Hydrogels for Biomedical Application

Carla Palomino-Durand, Marco Lopez, Frédéric Cazaux, Bernard Martel, Nicolas Blanchemain and Feng Chai

Supplementary data showing the cohesion of injected hydrogel through needle into PBS for 7 days

	0	30 min	1 h	6 h	1 day	7 days
3:0:3	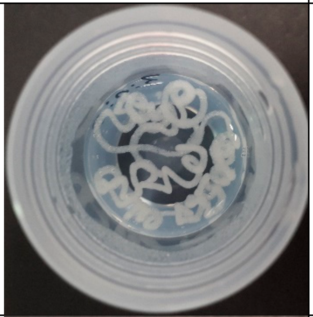	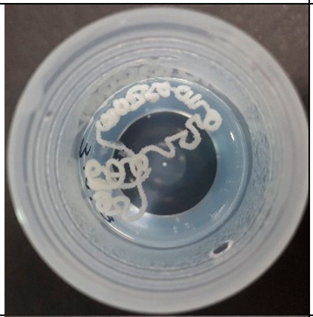	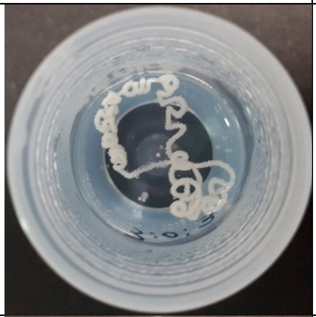	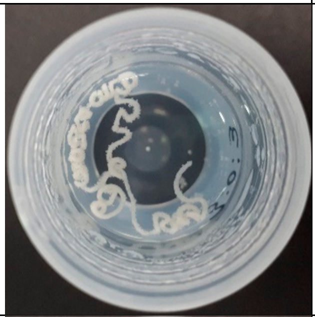	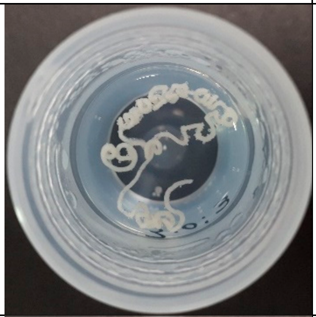	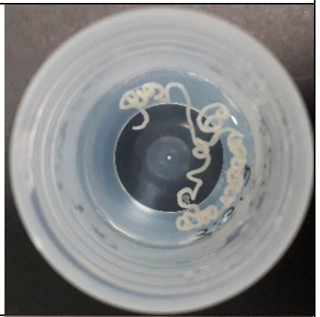
3:1,5:1,5	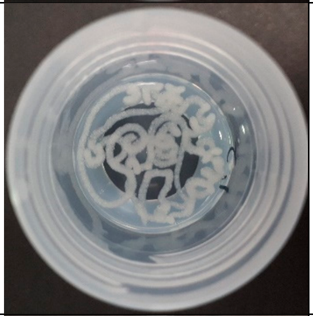	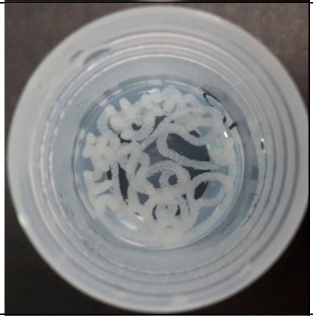	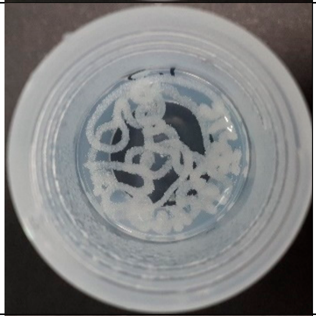	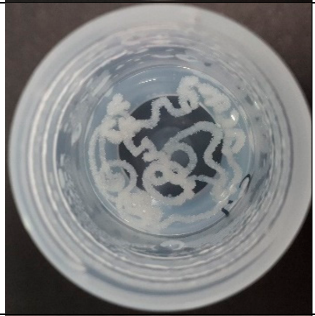	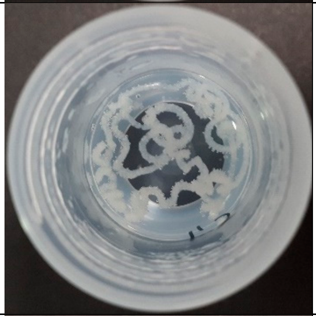	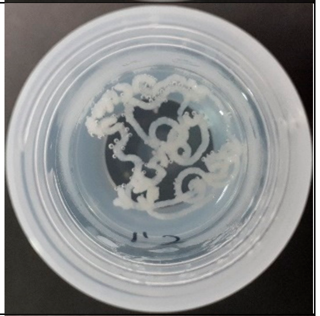
3:3:0	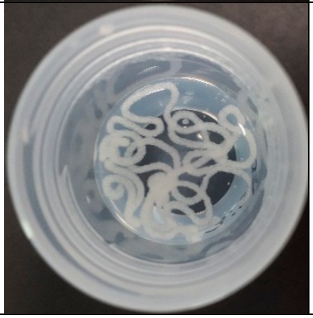	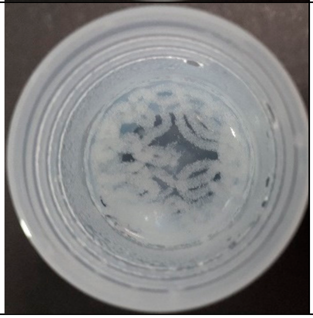	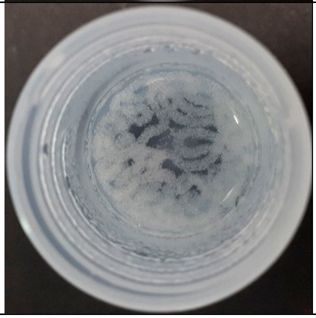	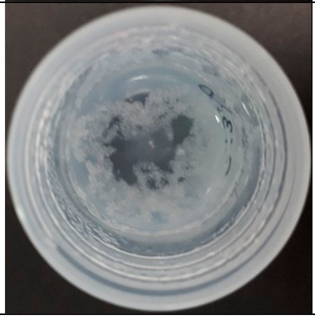	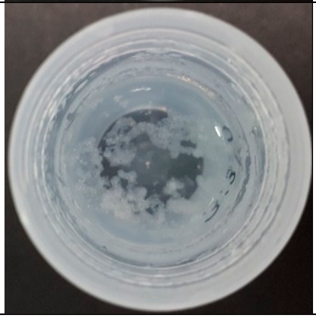	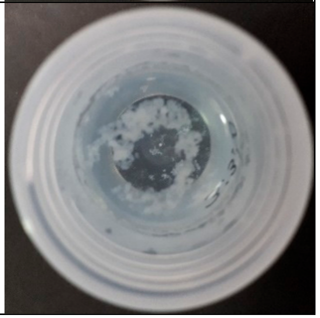
